# A Discussion of Building a Smart SHM Platform for Long-Span Bridge Monitoring

**DOI:** 10.3390/s24103163

**Published:** 2024-05-16

**Authors:** Yilin Xie, Xiaolin Meng, Dinh Tung Nguyen, Zejun Xiang, George Ye, Liangliang Hu

**Affiliations:** 1 Jiangsu Hydraulic Research Institute, Nanjing 210098, China; yilin.xie@jswater.org.cn; 2 School of Instrument Science and Engineering, Southeast University, Nanjing 211189, China; 3 Faculty of Engineering, Imperial College London, London SW7 2AZ, UK; 4 RWDI UK Ltd., Milton Keynes MK11 3EA, UK; tung.nguyen@rwdi.com; 5 Chongqing Survey Institute, Chongqing 401121, China; xiangzj@cqkcy.com; 6 UbiPOS UK Ltd., London EC2A 2BB, UK; george.ye@ubipos.co.uk; 7 Faculty of Architecture, Civil and Transportation Engineering, Beijing University of Technology, Beijing 100021, China; huliangliang@emails.bjut.edu.cn

**Keywords:** smart sensory network, cloud-computing, data strategy, digital twin, bridge monitoring

## Abstract

This paper explores the development of a smart Structural Health Monitoring (SHM) platform tailored for long-span bridge monitoring, using the Forth Road Bridge (FRB) as a case study. It discusses the selection of smart sensors available for real-time monitoring, the formulation of an effective data strategy encompassing the collection, processing, management, analysis, and visualization of monitoring data sets to support decision-making, and the establishment of a cost-effective and intelligent sensor network aligned with the objectives set through comprehensive communication with asset owners. Due to the high data rates and dense sensor installations, conventional processing techniques are inadequate for fulfilling monitoring functionalities and ensuring security. Cloud-computing emerges as a widely adopted solution for processing and storing vast monitoring data sets. Drawing from the authors’ experience in implementing long-span bridge monitoring systems in the UK and China, this paper compares the advantages and limitations of employing cloud- computing for long-span bridge monitoring. Furthermore, it explores strategies for developing a robust data strategy and leveraging artificial intelligence (AI) and digital twin (DT) technologies to extract relevant information or patterns regarding asset health conditions. This information is then visualized through the interaction between physical and virtual worlds, facilitating timely and informed decision-making in managing critical road transport infrastructure.

## 1. Introduction

Despite playing an indispensable role in global transportation, many large-span bridges suffer from structural damage and other issues. A very recent statistical analysis carried out by the RAC Foundation revealed that 3211 bridges in the UK were substandard as of the end of 2021, a 3.4% increase compared with those in 2020 [1]. As most of 2021 was under the COVID-19 pandemic lockdown in the UK, this rapid structural deterioration must have been caused by their natural degradation process. It is estimated that the cost to bring these substandard bridges back up to perfect condition would be GBP 1.16 billion and to clean up all the maintenance backlog on 70,944 bridges in the UK the cost would be GBP 5.44 billion [2]. There are much higher bridge inventories both in China and the US. The American Road & Transportation Builders Association (ARTBA) 2022 Report points out that 7% of its total 619,588 highway bridges are “structurally deficient” and 167.5 million daily crossings are on a total of 43,578 structurally deficient US bridges in poor conditions [3]. The estimated cost to retrofit all 224,000 bridges that need major repair work or replacement, including the 43,578 structurally deficient bridges, is USD 260 billion. By 2019, China had built more than 878,300 highway bridges, and 87% of these are medium and small-size bridges. More than 10% of these bridges are categorized as “structurally deficient”, which means that immediate retrofitting is required. Most of the long-span bridges in China (single span > 500 m for a suspension bridge and >300 m for a cable-stayed bridge) were constructed in the past 30 years and bridges built in the past 10 years usually have a built-in structural health monitoring (SHM) system due to regulations or laws.

Thanks to the technological development of the Internet of Things (IOTs), sensors, communication, and computer science, the structural health monitoring of long-span bridges in real time has gradually become practical and reliable. Many bridges installed SHM systems to reduce potential lockdown or damage risks worldwide [4,5,6,7]. An entire SHM system mainly includes a sensor module, a data acquisition and transmission module, a data processing and management module, and an assessment and notification module [8,9]. Correspondingly, many scholars have carried out much research on this topic, mainly including damage identification, Global Navigation Satellite System (GNSS) sensor applications, extreme event analysis, and SHM systems. GNSS technology has achieved significant progress in recent decades, which makes GNSS positioning a reliable sensor for the deformation monitoring of medium- and long-span bridges [10,11]. To improve positioning precision, the impact of GPS satellite and pseudolite geometry on structural deformation monitoring was analyzed [12]. Integrated GPS and triaxial accelerometers were deployed to detect the dynamic response [13]. A medium-span suspension bridge’s modal frequencies were attained using multimode GNSS positioning and multipath mitigation [14]. Luo proposed a component extraction method for the GNSS displacement signals of long-span bridges and validated it using the SHM data.

The SHM system should monitor the loading and structural parameters set by the bridge designer, assess the structural health status, guide the bridge-inspection activities, and, furthermore, provide advice on remedial works if damage is detected [15]. Structural modal parameters, such as natural frequencies, damping ratios, and mode shapes, are considered the critical assessment criterion for the structural health status. The use of a natural frequency from vibrating data as a diagnostic parameter in structural health assessment was discussed by Lee and Salawu [16,17]. An improved Bayesian modal identification approach, using scaled Fast Fourier Transform data for uncertainty quantification, was adopted to reduce the ambient uncertainties and enhance the reliability of modal tracking [18]. Bayesian spectral density was applied to address the uncertainty of mode extraction from the output-only response of a long-span suspension bridge to extract modal parameters from large data sets collected by the SHM system [19]. Deng proposed and proved the effectiveness of a method based on the correlation of the probability distribution of the quasi-static response data for damage identification since it is challenging to extract structural models or parameters that directly indicate structural damage [20]. Svensen et al. presented a novel hybrid structural health monitoring (SHM) framework for damage detection in bridges based on the finite element (FE) model and machine learning [21].

Extreme events, such as earthquakes, strong winds, and collisions, bring a great chance to watch and assess the bridge’s health status. An investigation of the dynamic properties of a long-span cable-stayed bridge was conducted during typhoon events based on structural health monitoring, and the relationship between modal parameters and environmental factors was revealed [22,23]. The operational modal parameters and seismic response of the 2160 m long Tsing Ma Suspension Bridge, when subjected to different earthquake events, were analyzed, which was beneficial to the seismic design of long-span bridges [5,24]. A ship collision incident happened at Jiangyin Bridge in May 2009, and the mode parameters were identified with GNSS measurements to evaluate the bridge condition [25]. A real-time artificial neural network model was proposed to investigate the real-time coupling relationship between multi-loads and bridge deformation, enabling the real-time prediction of bridge deformations [26].

**Figure 1:**
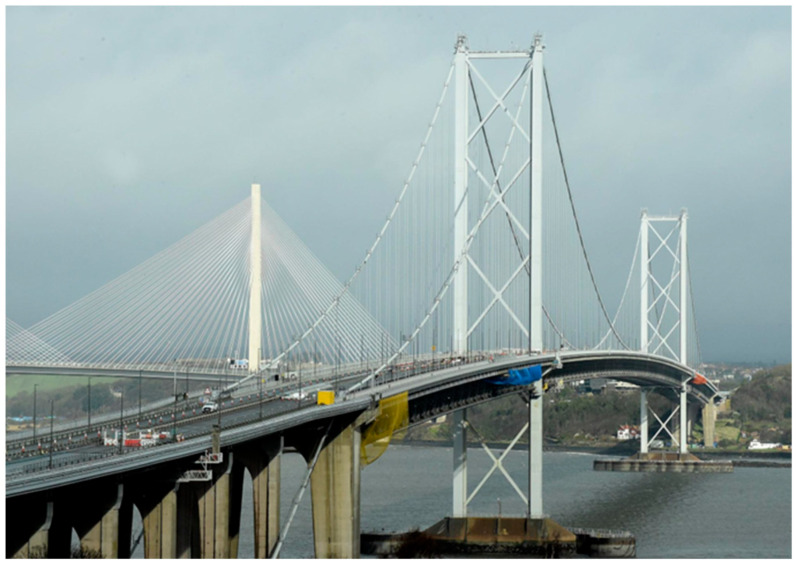
The Forth Road Bridge with the Queensferry Crossing as background (from: Edinburgh News).

From 2005, the corresponding author of this paper participated in, and then led, a series of episodic monitoring campaigns on one of the longest bridges in the UK, the Forth Road Bridge in Scotland [27]. With support mainly from the European Space Agency (ESA), a phased installation of a permanent monitoring system called GeoSHM started from 2014 [9]. The Forth Road Bridge was opened to traffic on 4 Sept 1964. The total length of the bridge is 2.5 km, with a main span of 1006 m (Figure 1). It was the world’s longest bridge outside the US, but now it ranks at number 44. The Forth Road Bridge is essential for the Scottish/UK economy. According to the former bridge master, closing one of its four lanes per day will cost GBP 650 K. The structural responses are dominated by temperature and traffic loadings and the bridge is susceptible to wind effects which cause excessive lateral movement and predicted over-stress under design wind loading. Regular repairs to the main components were carried out during its service lifetime of 58 years (https://www.theforthbridges.org/about-the-forth-bridges/forth-road-bridge/forth-road-bridge-maintenance/, accessed on 11 May 2024).

This paper uses the Forth Road Bridge in Scotland as an SHM example and the experience attained from the SHM systems erected on the bridges in China contributes enormously to the concepts and content of this paper. The overall focuses of this paper include the following:

- How to establish a cost-effective and smart sensory network for monitoring long-span bridges;
- How to develop an effective SHM data strategy to handle a large quantity of monitoring data from a high-rate sensory network;
- Analysis of the pros and cons of the cloud-computing technique for an SHM system; and
- A discussion of the integrated uses of digital twin (DT) and artificial intelligence (AI) for the smart monitoring of bridges.

We acknowledge that a concise conference version of this research was previously published by Meng [28]. However, our initial conference paper did not address how to diagnose structural health status. The present manuscript provides a thorough investigation, through data pre-processing and detailed analyses, for detecting structural changes and damages. Moreover, supplementary images and relevant information have been incorporated into the GeoSHM sensor network and data strategy, enhancing the article’s comprehensibility.

## 2. Smart Sensory Network for SHM

The monitoring objectives of the bridge owners, available budget, timeline, and, most importantly, the user demands together govern the overall design and configuration of an SHM system. This requires the SHM developers to sit down with bridge owners and listen to their most significant concerns in managing their assets. The selection of suitable sensor types, the determination of their optimal placement locations on the structure, and the design of the data transmission approaches are all essential factors to be considered in the initial stage of setting up a sustainable SHM system. To make this system adaptable to accommodate the changing demands of bridge owners and survive the harsh operational environment, smart sensors, and the network to connect them into an effective and efficient monitoring system, are widely used in current SHM systems. The smart level of the sensory system depends on the advancement of many key impact parameters (KPIs) or affecting factors such as a core chipset design, advanced materials and manufacturing skills, computing power, wireless or cabled communications, such as 5G/6G and fiber optic cables, etc.

In general, the monitoring parameters to be considered in a practical bridge monitoring system include the environmental parameters such as wind speed and its direction, temperature, humidity, rain, snow, ice, etc.; the spatial–temporal displacements (four-dimensional, 4D) of the structure, such as deflection and deformation, crack, fatigue, corrosion, etc.; and the forces on the structure, such as strain and stress and their 4D distributions. Since different bridge owners have their specific monitoring priority agenda, and since the size and complexity of each individual structure are different, the determination of the number and types of the sensors and their placements on the structure are unique. According to Xu and Xia [29] and Middleton et al. [30], the sensors used to measure these above three types of parameters include loading sensors for wind, earthquake, and traffic applied on the structures, the sensors for measuring structural responses such as displacement, strain, stress, etc., and the environmental sensors for measuring temperature, humidity, rain, ice, snow, corrosion, solar radiation, etc.

Nowadays, due to their unique advantages such as higher bandwidth, longer transmission distance, lower latency, and stronger security, optical fibers are extensively used for large quantities of monitoring data transmission in bridge SHM systems and various fiber optic sensors are developed to measure strain, temperature, dynamic and static vehicular weight, pressure, image, etc. Furthermore, the advancing and application of micro-electromechanical system (MEMS) sensors make bridge monitoring more accurate and affordable, significantly improving the density of the sensory network on the structure. The use of optical fibers and fiber optic and MEMS sensors have paved the way for integrating a digital twin (DT), analytic models such as the finite element model (FEM), and other advanced data sciences such as big data analytics and machine learning, etc., into the daily operation of SHM systems.

**Figure 2:**
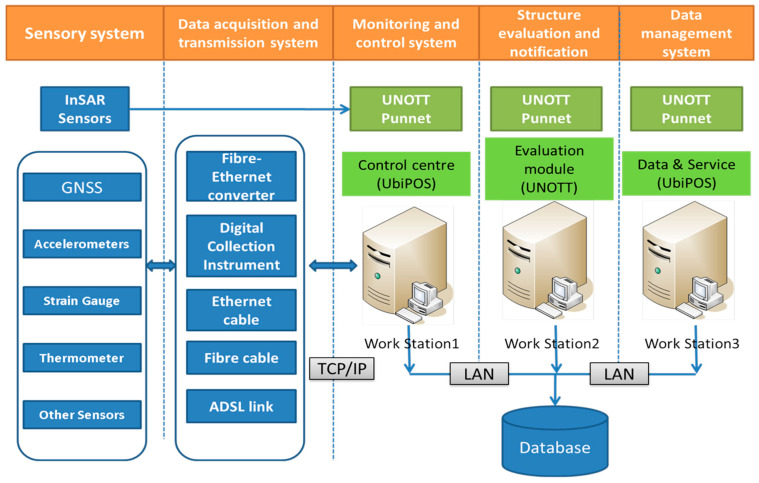
GeoSHM sub-systems and data flow.

Clearly, bearing this above development trend in mind and considering affordability, the Forth Road Bridge owner’s GeoSHM was designed as a phased, open, and scalable system [9,31]. The first phase was to prove the GeoSHM’s feasibility in fusing Interferometric Synthetic Aperture Radar (InSAR) regional inspection and in situ point-based monitoring with GNSS positioning and laying an optical fiber-based communication network to form high spatial–temporal monitoring [31,32]. The demonstration phase of GeoSHM development was more focused on densifying the existing monitoring footprint and developing a deep learning method to implement effective data mining, interpretation, and structural diagnoses [33]. The GeoSHM system consists of online and offline processing modules which play different roles: the online one for structural condition evaluation and the offline one for health and safety assessment. Figure 2 shows the main components of the GeoSHM sub-systems and how the GeoSHM in situ sensors are linked to the processing centers with the optical fiber network on the bridge and via the TCP/IP protocol through the Internet. The cabled-based network has been further upgraded using hybrid communication techniques.

**Figure 3:**
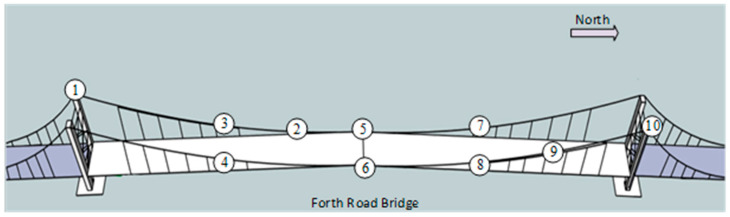
The sensor locations on the Forth Road Bridge (refer to Table 1 for the meaning of numbers).

In the GeoSHM system, different high-rate sensors are installed at various locations in the structure to gather the digital or analog signals of interest. Table 1 lists the sensors intended to be installed during its demonstration phase. Up to now, most sensors have been successfully installed and run on the bridge, but due to the pandemic replacing two corroded sensors at two quarter-span locations (SHM8 and SHM9) on the west side of the bridge, this was delayed. Figure 3 illustrates the current sensor types and locations.

Traditional sensing systems are mostly cable-based systems with monitoring nodes installed at essential locations in the structures and these systems are usually sparse and expensive. Great efforts are required to maintain the communication systems since the communication cables are more vulnerable to the environment than the sensors. With wireless communication, a large quantity of sensors could be installed flexibly and the overall monitoring systems could be more scalable and cost-effective.

Current SHM systems extensively use cable-based communications for data transmission due to the drawbacks in power supply, communication bandwidth, effective ranges, and signal interference due to complicated operational environments in wireless-based monitoring. Some wireless sensors operate on unlicensed transmission frequency bands such as 2.4 GHz; hence, output power limitations are imposed in different countries. For instance, this is 1 watt in the US and 0.5 watts in the UK. This affects both data transmission throughput and a valid range.

**Figure 4:**
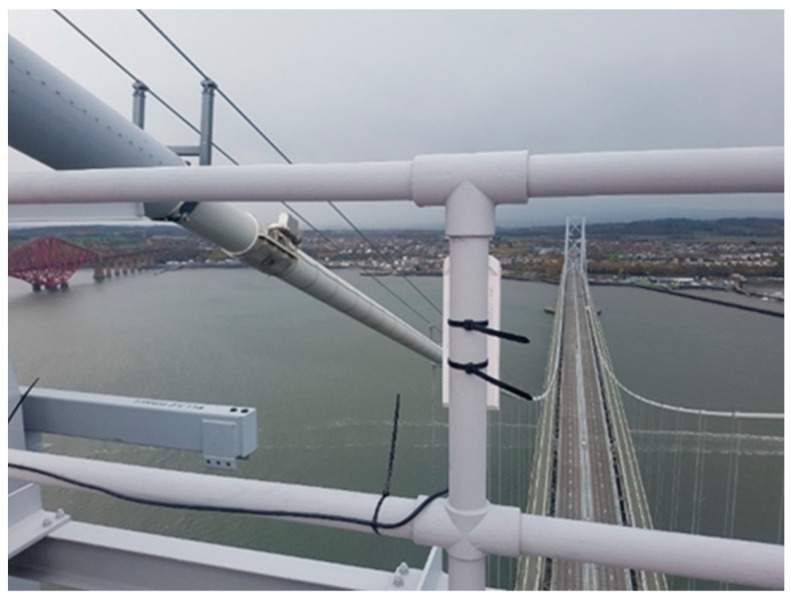
A TP-Link Wi-Fi transceiver point (CPE510) installed atop the northeast tower of the Forth Road Bridge.

When the optical fiber connection failed to transmit data due to the installation challenge and long transmission range limitation for a remote monitoring node in the GeoSHM project, a wireless communication method was tried. It turned out to be a very reliable and cost-effective solution. Therefore, the communication network used in the GeoSHM project is a combination of an optical fiber for most sensory sites and paired long-range Wi-Fi devices for a remote northeast (NE) supporting tower site. The NE monitoring node is the furthest point in the whole GeoSHM system and consists of a GNSS receiver, an anemometer, and a triaxial accelerometer. Figure 4 is the wireless communication set up of SHM5, which sits atop the NE tower of the Forth Road Bridge.

**Figure 5:**
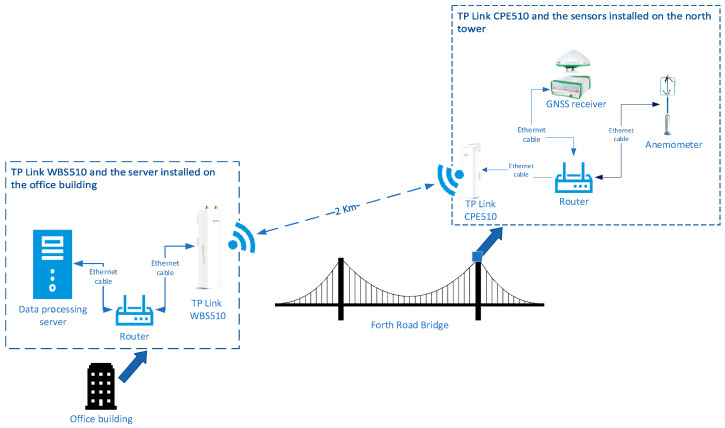
Data transmission between the NE tower and the office building using TP link device.

In this case, a pair of TP-Link’s Outdoor Wireless Base Station 510 (WPS510) and Access Point 510 (CPE510) was used to receive and transmit monitoring data. CPE510 is linked to the remote sensors of SHM5. WPS510 is installed on the roof of the FRB control center and connected to the Internet to stream the received data to the GeoSHM server to be processed. The nominal transmission range of these TP-Link devices is 5 km. Figure 5 demonstrates a schematic diagram of data transmission from the NE tower to the office building where the data processing server is located using a TP link device.

**Figure 6:**
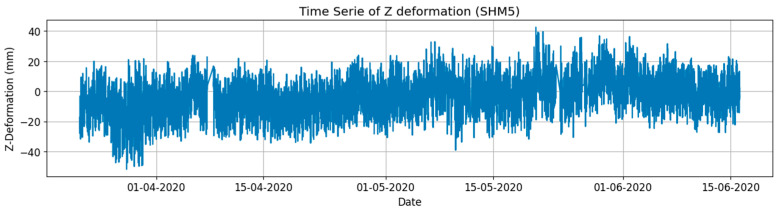
The time series of the vertical movement at SHM5 (10 Hz) in three months.

Figure 6 is the time series of the SHM5 vertical movement and an almost 100% transmission rate was achieved in the three-month period from March to June 2020 during the peak of the COVID-19 pandemic, which reflects the reliability of wireless communication for SHM. Additionally, it is noted that the transmission entails a time delay of approximately 10 ms, but the latency depends on various factors such as network conditions, distance between devices, and interference. The negligible data packet loss rate also indicates a stable signal.

By optimizing the placement of devices and the coverage range of signals, signal attenuation and interference can be reduced; selecting more advanced and efficient wireless communication technologies, such as LTE and 5G, can enhance the stability and speed of data transmission, thereby lowering packet loss and latency.

## 3. Development of an SHM Data Strategy

### 3.1. Data Strategy

Data strategy refers to the tools, processes, and rules that define how to manage, analyze, and act upon data. A data strategy helps different practitioners to make informed decisions based on the available data. It also helps to keep the data safe and compliant. According to Gartner’s definition, a data strategy is a highly dynamic process employed to support the acquisition, organization, analysis, and delivery of data in support of business objectives [34].

**Figure 7:**
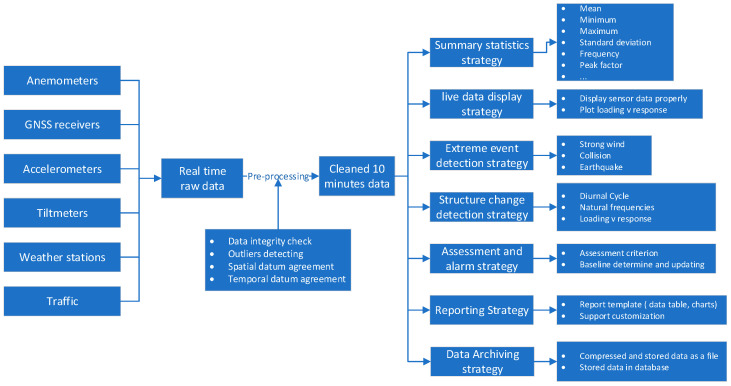
Data strategy for SHM.

Using the data-driven approach for the establishment of an SHM system, as illustrated in Figure 7, the aim of the development of an SHM data strategy is to provide the bridge owners and operators or service providers with appropriate bridge performance data and derived information to make informed bridge management decisions. The focus of the SHM data strategy should cover the procedures for data pre-processing and cleansing, real time data acquisition, data fusion from different sources, comparisons among real time data, historical ones and those from models, the detection of extreme events, and the identification of system changes. As digital twins, big data analytics, IoT, and AI are being applied more and more in the SHM of large infrastructure and efforts should be made in seamlessly plugging these into an updated SHM data strategy. As an example, the developed GeoSHM data strategy that has been utilized in guiding the development of the GeoSHM system comprises five interlinked components:

(1) Data acquisition and pre-processing. All the raw measurements and the corresponding derived time series should refer to common spatial–temporal data such as those defined by the Global Navigation Satellite System (GNSS) and using multi-GNSS, including BDS, becomes indispensable when a precise spatial–temporal datum is required for life-cycle infrastructure monitoring [35]. Data acquisition rates and smart triggers for sensor controls should be determined, validated, and operational. Also, an appropriate bridge coordinate system (BCS) should be defined, where its *X*-axis coincides with the main axis of the bridge (longitudinal), its *Y*-axis is perpendicular to the *X*-axis in the horizontal plane (lateral), and the *Z*-axis points in the vertical up-direction. All the global, local, and body-framed coordinate systems of the monitoring sensors should be linked to the BCS through rigid coordinate transformations. Outliers and gaps in the acquired data sets should be detected and removed before further processing.

(2) Data architecture and integration. A data architecture is devised and used to guide the data archive and storage process. Immediately after data pre-processing, the cleaned data sets will be pushed to the dedicated SHM Cloud. Further data processing, including data fusion and mining from summary statistics of whole data sets, will be performed on a dedicated processing engine. A heterogeneous database structure is used to store all the data in the same Cloud.

(3) Data storage and technology. The GeoSHM users have access to the layered live data stream, historic data, structural health status report, and so on, according to their roles, which include bridge masters, engineers, researchers, and the general public. This part of the work is to set data storage and access rules (raw data vs. processed ones, and duration for keeping this large quantity of raw measurements) and the kinds of media for storing these data. Solutions are compared against KPIs such as cost, performance, ease of access, etc.

(4) Data insight and analysis. A direct comparison of the short-term statistics with the historical data will be made using statistical control charts. Bridge performance data will be presented on a chart relating the maximum displacement (or other variables) to incident loading (traffic, wind, or combined). Live bridge performance data will be presented as a time series with thresholds based on historical data analysis. These will include how to use finite element models, AI, and DT to enhance structural health condition assessments.

(5) Data governance, privacy. Setting up of the rules regarding who is the owner and who is responsible for the data, who can access the data, and how they can access them.

### 3.2. Outlier Removal by Hampel Filter

The emergence of outliers represents a crucial aspect of data processing strategies in structural health monitoring systems. Due to equipment manufacturing and external environmental factors, the data collected by sensors may sometimes contain outliers. To ensure the accuracy of subsequent data analysis, outlier removal is the first step in data processing. In the GeoSHM project, Hampel filtering is applied to detect and remove outliers in sensor data.

The Hampel filter works by analyzing the data and identifying and removing outliers that deviate from the overall data distribution, resulting in more robust estimation results. The principle of the Hampel filter is based on the concepts of median and Median Absolute Deviation (MAD). It involves sliding a window over the data and calculating the absolute deviation of data points from the median, the marking of data points beyond a pre-set threshold (usually three times MAD), and replacing these outliers with the median or other appropriate estimated values. For a data set *X*_1_, *X*_2_, …, *X_n_*, *MAD* can be calculated using the following formulas:

(1) $$\tilde{X}=median(X)$$

(2) $$MAD=median\left(\left|X_{i}-\tilde{X}\right|\right)$$

The Hampel filter has strong robustness in handling outliers, effectively dealing with anomalies in the data. Furthermore, it can, to some extent, preserve the original features of the data compared to other filter methods. However, the Hampel filter requires setting a window length and threshold beforehand, and the choice of these significantly impacts the filter effect. Improper selection may lead to the filtering out of important information or the retaining of too many outliers.

**Figure 8:**
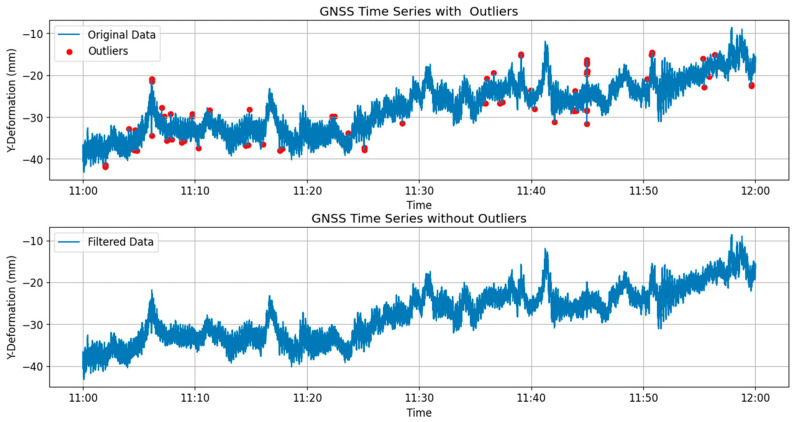
Original data and the filtered data for GNSS time series.

**Figure 9:**
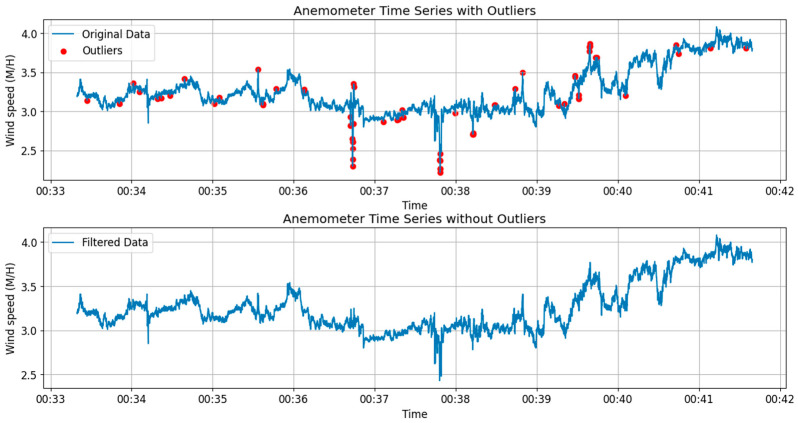
Original data and the filtered data for wind speed time series.

Figure 8 illustrates the GNSS time series plot comparing the original data set with the outlier-removed data, focusing on the *Y*-axis direction at the mid-span, where the sensor ID is SHM2. Meanwhile, Figure 9 showcases the time series plot derived from data captured by the ANE2 anemometer, positioned atop the north tower.

### 3.3. Data Mining for Structural Health Assessment

The data mining module exploits the GeoSHM database using a combination of advanced model-free methods (surrogate models and pattern recognition) and model-based methods (finite-element models) to extract information regarding the health status of bridges. The data mining module consists of six inter-linked sub-modules to implement structure diagnosis:

i. Creation of training data for damage detection;
ii. Analysis of baseline performance;
iii. Level 1 diagnosis: System change detection;
iv. Level 2 diagnosis: Damage detection;
v. Level 3 diagnosis: Reliability assessment;
vi. Updating process.

**Figure 10:**
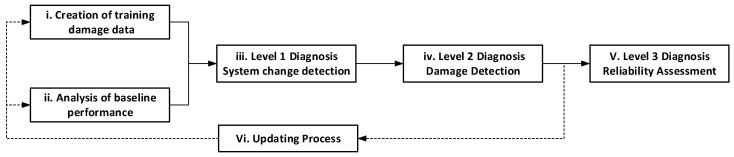
Dependence between sub-modules.

The relationship between the six sub-modules is shown Figure 10.

**Figure 11:**
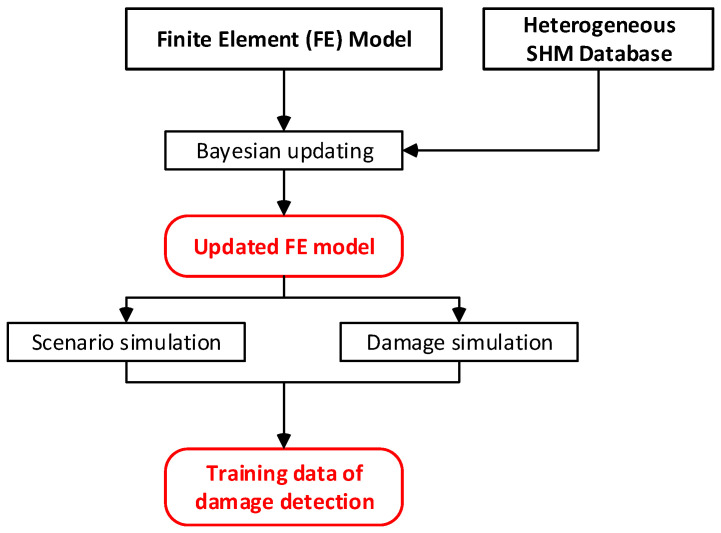
Sub-module (i): Creation of training data for damage detection.

**Figure 12:**
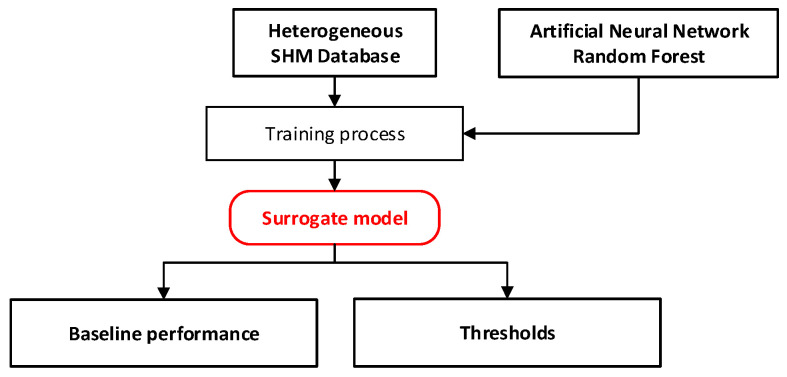
Sub-module (ii): Analysis of baseline performance.

Sub-modules (i) and (ii) create additional data and fundamental models which will be utilized in subsequent sub-modules to diagnose the health status of the bridge; Figure 11 and Figure 12 show the flowcharts of the sub-modules (i) and (ii).

**Figure 13:**
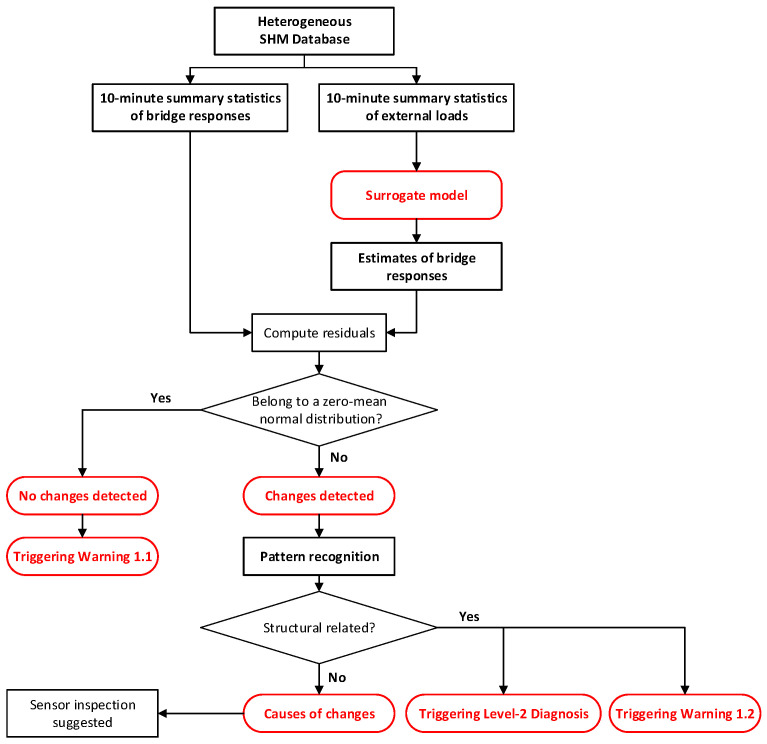
Sub-module (iii): Level 1 diagnosis of system change detection.

**Figure 14:**
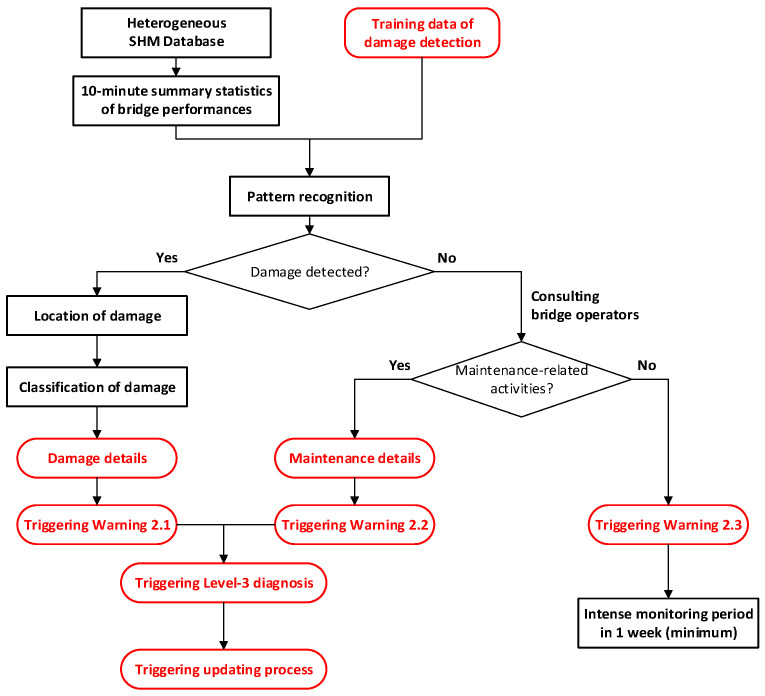
Sub-module (iv): Level 2 diagnosis of damage detection.

Sub-modules (iii) and (iv) design five warnings for its various stages, each tailored to detect specific structural health concerns. These warnings enable real-time feedback, ensuring the continuity and efficiency of the monitoring process. They pinpoint potential risks, informing critical maintenance and management decisions, and thus enhancing bridge safety. Figure 13 and Figure 14 demonstrate the flowcharts of the sub-modules (iii) and (iv).

- Warning 1.1: No abnormal behaviors detected.
- Warning 1.2: Structural-related changes detected.
- Warning 2.1: Structural damages detected.
- Warning 2.2: Changes due to maintenance activities.
- Warning 2.3: Changes are not diagnosed. Intense monitoring is required.

**Figure 15:**
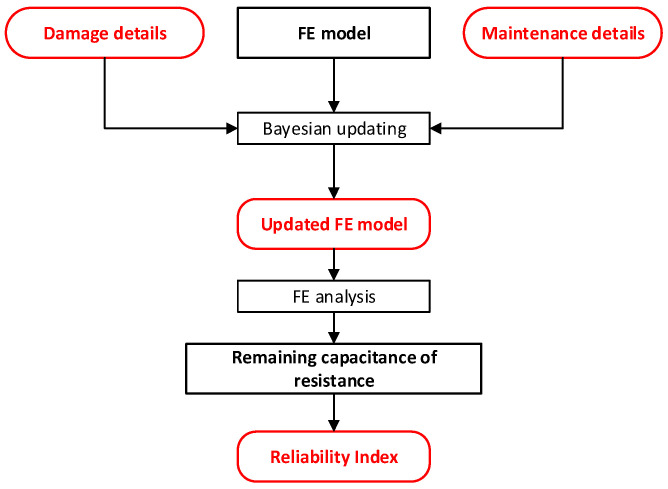
Sub-module (v): Level 3 diagnosis of reliability assessment.

Sub-module (v) is level 3 diagnosis for reliability assessment, which leverages a FE model, damage details, and maintenance records to assess the reliability index of a bridge. Initially, the damage or maintenance specifics obtained from Sub-module (iv) are employed to update the FE model using a Bayesian updating framework. Subsequently, an FE analysis is conducted on the updated model to evaluate the residual resistance capacity of the bridge. This evaluation is then translated into a reliability index by comparing it against the current loading conditions and stress levels on critical bridge members. The effectiveness of this sub-module is contingent on the availability of accurate and timely damage and maintenance details. See Figure 15.

Sub-module (vi) is an updating process with the core function of recalling sub-modules (i) and (ii) to update the FE model and regenerate a surrogate model, including the knowledge of structural changes due to damage or maintenance activities. Subsequently, a new set of training data for damage detection, baseline performances, and thresholds for change detection will be created. Generally, adjusting the threshold typically entails updating both the finite element model and the surrogate model using recent sensor data collected over a quarter or a year, alongside structural maintenance activities information.

Handling missing parts in measured data is a common challenge in data analysis. To address this issue, various methods can be employed. One common approach is to use data imputation techniques to estimate the missing values based on the characteristics of existing data. For example, linear interpolation, polynomial interpolation, or machine learning-based methods can be used for imputation. Another approach is to utilize statistical distributions and models to predict the missing values. For instance, probability distributions of missing data can be estimated based on the statistical features of the data, and these distributions can be used to generate random samples for missing values. Additionally, multiple imputation methods can be considered to generate multiple plausible data sets, capturing the uncertainty associated with missing data. By carefully considering the characteristics of the data and the requirements of the analysis, selecting appropriate methods for handling missing data can improve the accuracy and reliability of data analysis.

When it comes to evaluating the quality of monitoring data, several considerations need to be taken into account. Firstly, data completeness should be assessed by evaluating the proportion of missing data in the data set and the pattern of missingness. Secondly, the accuracy and precision of the data should be evaluated by comparing them to known reference standards or other independent data sources. Furthermore, consistency checks should be performed to ensure the consistency of data across different time points and locations. Finally, the stability and reliability of the data should be considered to assess its stability and reproducibility under different conditions. By taking into account these aspects, a comprehensive method for evaluating the quality of monitoring data can be established, providing a reliable foundation for data analysis.

## 4. Cloud-Computing Technique for SHM

As defined by Ray [36], cloud-computing is the on-demand availability of computer system resources, especially data storage, which is called cloud storage, and computing power, without the direct active management of IT facilities by the users. The advantages and disadvantages of using cloud-computing for an SHM system are listed below.

The advantages of using a cloud-computing platform are as follows:

(1) Low-cost and easy to use. Basically, a cloud-computing company provides the platform to make, for example, physical resources, processing, memory, storage, and network capabilities virtually, which can help clients build their systems easily and quickly at a low cost.
(2) Scalable computing resource. The cloud company provides a service with the capability to be expanded and contracted as required. Take the GeoSHM system as an example; fewer resources need to be purchased at the initial stage, and the computing performance and storage space can be gradually increased as the amount of data increases in operation.
(3) Easy to maintain. Because there is no physical infrastructure, there is no need for dedicated and skilled personnel to perform regular maintenance on the equipment. Cloud vendors have already carried out all this for the clients.
(4) Provide hacking prevention and data security service. Cloud vendors provide different levels of hacking prevention and data security services according to customer requirements, which make the whole system more reliable.

The disadvantages of using cloud-computing include the following:

(1) Data security and privacy. Service providers have the opportunity to directly access the data, which poses potential risks to data security and privacy.
(2) Migration of data and services. Transferring a large quantity of data/services from an old provider to a new one can be very painful and cumbersome if the SHM user wishes to switch to another provider.

The traditional way of building a structural health monitoring platform starts from purchasing computer workstations and servers, routers, gateways, and other hardware equipment, software tools, etc. Due to the lack of maintenance facilities, such as a UPS backup power supply, dual network devices, a standard air-conditioning room, 24/7 professional maintenance personnel, etc., this may cause data loss because of power failure, network disconnection or even system failure, etc. The data loss statistics using the traditional data storage method in a server at the University of Nottingham (UNOTT) from 2017 to 2019 are listed in Table 2.

**Figure 16:**
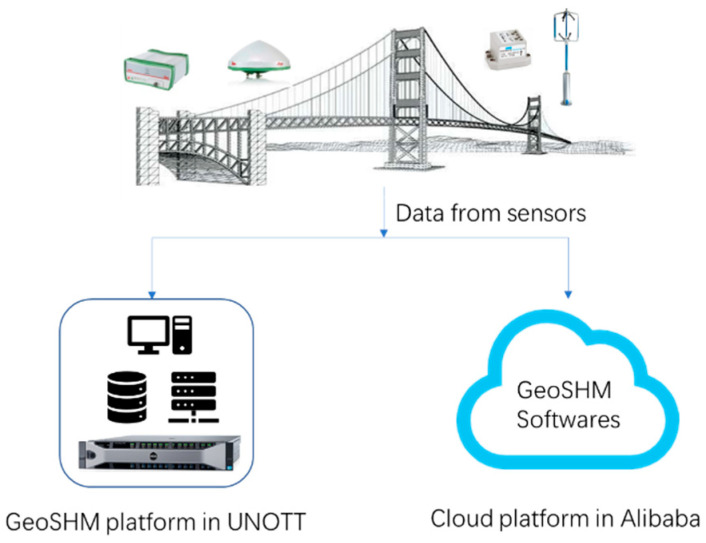
Hybrid SHM processing platform using Ali Cloud and a local server UNOTT.

From July 2020, the architecture of the GeoSHM system has been changed and the same data were sent to the Cloud, the physical servers of which are located in London and provided by Alibaba Cloud Ltd. (Hangzhou, China). Figure 16 is the hybrid computing architecture of the GeoSHM system. After a period of debugging and optimization, the entire system was completely transferred to the Alibaba Cloud. There was no data loss caused by the Ali Cloud server.

The Ali Cloud SHM server mainly includes the following three running software modules:

(1) The data receiving and management software module. This is used for data receiving, cleaning, and pre-processing and for storing relevant statistical data and original observation measurements according to the designed SHM data strategy.
(2) Data processing and analysis management software modules. This is used for estimating the SHM parameters and model update driven by AI, the dynamic adjustment of thresholds, the real-time health assessment of bridge structures, and warning services.
(3) Web-based network client system. It provides real-time data queries and statistics, historical data comparison and analysis, real-time health status queries, and other related SHM functions.

In addition to this, the Ali Cloud provides a database and file storage service. According to the data storage strategy, all of the sensor raw data, cleaned data, intermediate results, the final assessment, and other management data are stored in the form of files or databases.

Therefore, addressing the current shortcomings in cloud-computing related to data security and privacy, as well as data and service migration, the following proposed solutions are presented. To enhance data privacy and security, it is paramount to reinforce data encryption measures, ensuring security during data transmission and storage processes. Strict access control and identity verification mechanisms must be implemented to restrict access to sensitive data. Additionally, selecting a reputable cloud service provider and, if necessary, executing a distinct data privacy and security agreement are crucial steps to safeguarding data. To alleviate the complexities and costs associated with migration, it is vital to develop a comprehensive data migration plan at the outset of cloud platform design. Utilizing standardized data formats and interfaces can streamline the migration process. Moreover, opting for service providers that support open interfaces and leveraging third-party migration tools and services can simplify migration, ensuring the continuity and stability of the system.

## 5. Digital Twin (DT) and Artificial Intelligence (AI) for Smart Monitoring of Bridges

**Figure 17:**
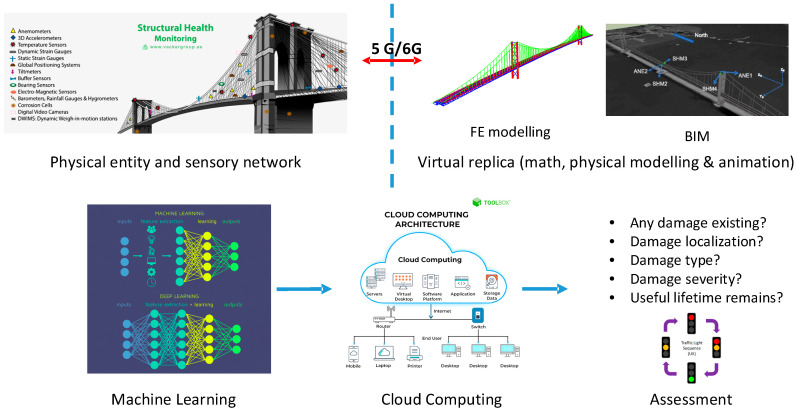
Digital Twin (DT) and Artificial Intelligence (AI) for smart monitoring of bridges.

In the SHM of bridges, a digital twin intends to build a virtual model that uses the real-world heterogenous data sets gathered from an operational bridge as its input for enhancing asset management process. Once precisely constructed, the DT can be used to simulate and predict the possible impacts of certain environmental and loading effects using monitoring data sets as the DT inputs. Linking the DT and artificial intelligence, especially using deep learning and transfer learning, is essential and a current development trend in implementing smarter, safer, and higher-quality infrastructure inspection regimes (Figure 17).

The method for real-time bridge condition assessment utilizing Digital Twin technology comprises the establishment of a Digital Twin model, the deployment of sensor networks, data fusion, real-time simulation and analysis, and fault diagnosis and predictive maintenance. Firstly, the Digital Twin model accurately reflects the geometric structure, material properties, and environmental influences of the bridge. Subsequently, the key parameters of the bridge, such as the vibration, strain, and temperature, are monitored in real-time through sensor networks. The collected sensor data are then fused with other data sources, such as meteorological and traffic data, to obtain comprehensive information on the bridge’s operational status. Next, the collected data are subjected to real-time simulation and analysis using the Digital Twin model to evaluate the current condition of the bridge and predict potential faults. Finally, maintenance strategies are formulated based on the analysis results to ensure the safe operation of the bridge. Throughout this process, measures are recommended to ensure model accuracy, deploy advanced sensor technologies, and develop efficient data processing algorithms to enhance the efficiency and accuracy of bridge condition assessments.

It is extremely useful while seeking timely, robust, accurate, and cost-effective solutions to address the many overdue bridge inspections around the world. This new way of inspecting includes the integration of smart sensors or other field data acquisition, secured data transmission via cabled and 5G/6G or even LEO satellite broadband comms, building high-definition (HD) 3D or 4D models of the assets with DT techniques, and using both AI and analytic models such as FEM to process, analyze, and visualize huge monitoring data. Pairing DT with AI that uses the data from a smart sensory system on the structures as its inputs, and the high-speed data communications, provides great opportunities for conducting instant interactions among the physical-world features and the cyber-world models to maximize monitoring effects [37].

Artificial Neural Networks (ANNs) have been successfully applied in many fields including pattern recognition, connected and autonomous vehicles, civil engineering, public security, etc. [38]. In the area of SHM, ANNs are one of the most common methods for studying the relationship between the bridge responses and loading factors. Bayes is used to determine the conditional probability, which is the likelihood of an outcome occurring, based on a previous outcome having occurred in similar circumstances. Compared with other AI algorithms, Bayes-based Artificial Neural Networks (BANNs) have the following characteristics:

- A single-layer feed-forward neural network;
- A quicker learning process;
- Better generalization;
- The quantification of the uncertainty of predictions.

**Figure 18:**
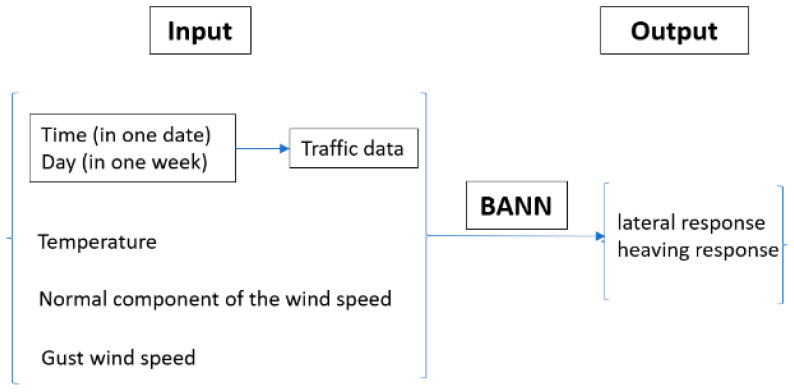
Input and output of the BANN algorithm.

The last character is extremely important, since sensors on the structures bear errors or outliers which are detrimental to the success of structural condition assessments. In the GeoSHM data analytics toolbox, a BANN is employed to generate a non-linear regression model to estimate time-dependent lateral and heaving responses with respect to variations in wind, temperature, and traffic. The inputs and outputs of the regression model are 10 min average statistics and the use of the BANN for monitoring data analysis is described in Figure 18.

The formula of the Bayesian inference is as follows:

(3) $$p(\theta |D)=\frac{p(D|\theta )\cdot p(\theta )}{p(D)}$$

where

*p*(*θ*) is the prior probability of a parameter *θ* before having seen the data.
*p*(*D*|*θ*) is called the likelihood and is the probability of the data *D* given *θ*.
*p*(*θ*|*D*) is the posterior probability of *θ* given the data *D*.

Instead of considering a single answer to a question, Bayesian methods allow us to consider an entire distribution of answers which can solve issues like regularization (overfitting or not), model selection/comparison, and cross-validation data set separation.

**Figure 19:**
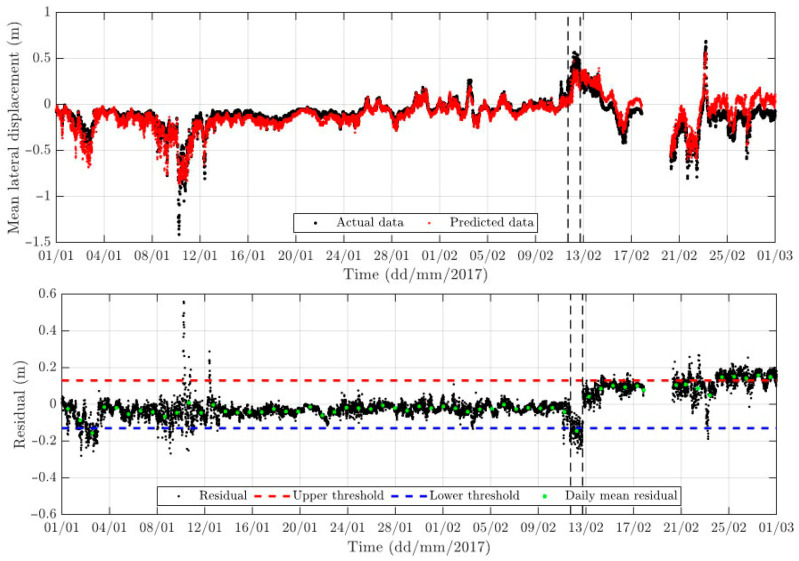
Structure change detection based on the BANN of the Forth Road Bridge.

Empowered by the BANN, a new rolling assessment and updating approach has been developed for the GeoSHM project to detect structural changes in the Forth Road Bridge, as shown in Figure 19. The first row in this figure is a time series comparison between the measured mean lateral movements and those predicted using the BANN. The second row includes the difference in the two time series in the comparison (called residual), the upper and lower thresholds determined using at least one year’s monitoring data (loading and response), and the daily mean residual, which is used to assess the structural changes. It is apparent that, after 13 February 2017, the daily means are either close to or moved out of the set thresholds. A further investigation reveals that, on this date, the maintenance team had completed the truss end link repair at the northeast main span, which changed the dynamic characteristics of the bridge (https://www.theforthbridges.org/news-and-blogs-from-the-forth-bridges/truss-end-links-repair-one-year-on/, accessed on 11 May 2024).

## 6. Conclusions

In this paper, the authors discussed four aspects in establishing structural health monitoring systems for long-span bridges. There is a wide range of smart sensors that could be used to monitor loading, response, and environmental factors; however, on the journey to establish digital twins to support timely and informed SHM decision-making, both the sensors’ quality and density need to be improved. Wireless communication might be a good option when other means of data transmission fail to work, and authors have demonstrated how long-range Wi-Fi could bridge this gap. Large and high-quality monitoring data are the basic requirements for an SHM system and to manage these data; a data strategy is essential for building a successful and long-lasting SHM. Using the GeoSHM project as an example, the authors presented in detail what should be covered in data strategy development, especially regarding how to detect the structural status change and damage. This paper also analyzed the pros and cons of cloud-computing techniques for an SHM system. Based on the authors’ experience, cloud-computing represents a significant advancement in SHM system architecture design. Finally, an introduction of the integration of DT and AI for smart bridge monitoring is followed by a discussion of the successful application of the BANN model in detecting structural changes in the Forth Road Bridge and its associated benefits. In summary, integrating a smart sensory system, IoT, a data strategy, cloud-computing, DT, and AI will greatly benefit the monitoring and inspection work of long-span bridges.

## Figures and Tables

**Table 1:** A complete list of the sensors planned in the Phase Two development of the GeoSHM system.

**No**	**Location**	**Instruments**	**ID**
1	Top of the SW tower leg	GNSS	SHM4
		Anemometer	ANE1
		Accelerometer	ACC1
		Tiltmeter	TLT1
1-1	Mid-height of the SW tower leg	Tiltmeter	TLT2
2	PP87 SW (3/8 of the main span)	Accelerometer on cable	ACC2
		Accelerometer on deck	ACC3
3	PP73 SW(NAV channel)	GNSS	SHM8
4	PP73 SE (NAV channel)	GNSS	SHM7
		Accelerometer	ACC3
5	PP101W (Mid span)	GNSS	SHM2
		Anemometer	ANE2
6	PP101E (Mid span)	GNSS	SHM3
		MET station	MET1
		Accelerometer	ACC5
7	PP73NW(NAV Channel)	GNSS	SHM9
8	PP73NE(NAV Channel)	GNSS	SHM6
		Accelerometer	ACC6
9	PP59 NE (1/8 of the main span)	Accelerometer on cable	ACC7
		Accelerometer on deck	ACC8
10	Top of the NE tower leg	GNSS	SHM5
		Anemometer	ANE3
		Accelerometer	ACC9
		Tiltmeter	TLT3

**Table 2:** Data loss statistics from 2017 to 2019.

Reason	Time Frequency	Days of Data Loss
Network disconnection	5	23
Power failure	6	9
System failure	5	20
Total	16	52

## Data Availability

Dataset available on request from the authors.
